# A shotgun approach to explore the bacterial diversity and a brief insight into the glycoside hydrolases of Samiti lake located in the Eastern Himalayas

**DOI:** 10.1186/s43141-022-00444-y

**Published:** 2022-12-05

**Authors:** Aditi Rai, Shyama Prasad Saha, Toral Manvar, Arindam Bhattacharjee

**Affiliations:** 1 Department of Microbiology, University of North Bengal, P.O. NBU, District Darjeeling, West Bengal, Pin-734013 India; 2 Xcelris Labs Ltd, Ahmedabad, Gujarat 380006 India

**Keywords:** Diversity, Glycoside hydrolases, Samiti Lake, Himalayas, Psychrophilic, Psychrotrophic

## Abstract

**Background:**

The Himalayas have always been an enigma and, being biodiversity hotspots, are considered extremely important from an ecological point of view. Recent advances in studies regarding high-altitude lakes have garnered relevant importance as these habitats could harbor potential psychrophilic and psychrotrophic microbes with bio-prospective applications. Contemplating the above scenario, the present study has been undertaken to understand the diversity and the functional capacities of the microbes thriving in this lake.

**Results:**

In our present study on Samiti Lake, the abundance of Proteobacteria as the major phylum was seen in both the soil and water samples. Incase of the ABSLW (water) and ABS1 (soil) sample, 148,066 and 239,754 predicted genes, were taken for functional analysis. The KEGG analysis showed that ABSLW and ABS1 had 122,911 and 160,268, genes assigned to KO terms respectively. Whereas in case of COG functional analysis, 104,334 and 130,191 genes were assigned to different COG classes for ABSLW and ABS1 respectively. Further, on studying the glycoside hydrolases, an abundance of GH13, GH2, GH3, GH43, and GH23 in both the soil and water samples were seen.

**Conclusion:**

Our study has provided a comprehensive report about the bacterial diversity and functional capacities of microbes thriving in Samiti Lake.  It has also thrown some light on the occurrence of glycoside hydrolases in this region, as they have numerous biotechnological applications in different sectors.

**Supplementary Information:**

The online version contains supplementary material available at 10.1186/s43141-022-00444-y.

## Background

Culture-independent approaches to studying microbial diversity in extreme environments have increased significantly because of their bio prospective aspects [1, 2]. These unexplored extreme environments and their functional diversity studies could facilitate various potential bio-prospective solutions to environmental concerns [2]. Recently, ecology and biodiversity relationships with bacterial communities via environmental sampling and the next-generation sequencing technologies have been at the forefront of research [3]. Since most microorganisms are still difficult to cultivate hence, culture-based methods have been found to be insufficient for determining the diversity of microbes [4]. Exploring the microbial diversity via metagenomics has been a boon to science as, it has promoted and enabled a better understanding of the unexplored and extreme realm and diversified our knowledge about microbial adaptations and their interactions in unexplored areas [5, 6]. Numerous metagenomics studies have been carried out in aquatic environments [7, 8]. Diverse metagenomics studies on the psychrophilic Pangong Lake [9], Upper Mississippi River (Minnesota) [10], and Amazon Basin freshwater lakes [11] have recently been reported. The metagenomics methodology has thus paved the way through which novel gene sequences, and metabolic pathways of unculturable microorganisms have been identified as has been elucidated by some recent works [12].

High-altitude lakes are exposed to extreme environments like low nutrient conditions, UV radiation, and low temperatures [13, 14]. Furthermore, these high-altitude lakes are harder to access than low-altitude lakes due to their distant location [15]. Additionally, the mountain ecosystems show altitudinal gradients and adverse environmental factors [16–18]. Reports suggest that although the diversity of flora and fauna shows an inverse relationship with increasing altitude, it  may not apply to microbes [19, 20]. The drastic seasonal shifts in the physiochemical properties of the soil, climatic changes in altitude gradient and fluctuating subzero temperatures are characteristics of the Himalayan range [1, 21]. The Himalayas are biodiversity hotspots for different flora and fauna, ranging from orchids and rhododendrons to Himalayan tahr, Red panda, Himalayan Musk deer, Black Eagle, Tibetan Partridge [22, 23] etc. Likewise, this region may also hold a great promise of untapped potential for microbial diversity. Recent metagenomics research on Manikaran Hot Springs [24], frozen soil of the northwestern Himalayas of Jammu and Kashmir [25], and high-elevation Himalayan glacial lakes (Parvati kund) imply that these Himalayan regions are undergoing exploration [26]. Further, the Eastern Himalayas provide a plethora of high-altitude lakes [27] and such regions could harbor numerous psychrophilic and psychrotolerant microorganisms [28] that could be the source of numerous cold-adapted enzymes [29]. Psychrophilic enzymes have a wide range of documented uses, including stone washing [30], bioremediation, biotransformation, biomedical [31–33], and molecular biology applications [34, 35]. Reports of bio-augmentation with psychrophiles have also been shown to improve the biodegradation of recalcitrant substrates [36]. One such enzyme, glycoside hydrolases (GH), has an increased potential for complex carbohydrate deconstruction [37]. Glycoside hydrolases like cold active cellulases [38], amylases [39, 40], and β-galactosidases [41, 42] have found to be advantageous in a variety of industries like that of detergents, cosmetics, food, textiles, and bakery [43].

Sikkim is a Himalayan state  situated in the northeastern part of the Indian territory, close to the Eastern Himalayas [44]. Samiti Lake, is a glacial lake in Sikkim that sits at an elevation of approximately 4200 to 4300 m (13,700 ft.) [45]. The high elevation of the lake and the cold temperatures prevailing in this region, are apt for the detection of diverse microflora  having metabolic pathways that they use as a survival tactic in such extreme environments. It is in this regard that the present study was undertaken, to have an insight into the microbial community thriving in this region and to unearth the presence of glycoside hydrolases as they are presently being used extensively in the biotechnological industries.

In the present study, diversity analysis was done using Kaiju, Cognizer was used to obtain functional annotation against the COG, KEGG, Pfam, FIG, and GO databases. Ecological distance matrices were computed and employed to determine the diversity of the species. Further, to study the GH families, the CAZy database was employed.

## Materials and method

### Collection of environmental sample

Soil samples (ABS1) (100 g) at depths of approximately 10 to 12 cm, beneath the soil surface from Samiti Lake in sterile plastic and glass containers, and water samples (ABSLW) of 2.5 L were collected from three different points in the lake at 1-m depth. After careful homogenization of the soil, they were stored at −20° C. All of the samples were further transported to Xcelris labs for DNA sequencing. The samples were pooled before processing for DNA isolation.

### Physicochemical parameters

The physicochemical properties of water like pH and conductivity were analyzed using Eutech’s Cyber Scan PCD650 (a handheld waterproof meter). The soil organic carbon was estimated by chromic acid method proposed by Walkley and Black [46], and the Nitrogen content was measured by the Kjeldah method [47].

#### Isolation of DNA and library preparation

For metagenomic analysis, the Xcelgen Soil DNA isolation kit was used to isolate the DNA, and 0.8% agarose gel was used to detect the DNA (loaded 3 μl). Covaris was used to shear the DNA. Further, HiFi PCR Master Mix was used to amplify the fragments. The paired-end sequencing library was performed using NEB Next Ultra DNA library Prep Kit [48]. The size of libraries as determined by the Agilent bio analyzer was 470bp and 475bp for ABS1 and ABSLW samples, respectively. Further, the libraries have been submitted in NCBI with accession numbers SAMN13671136 and SAMN13671135. Illumina HiSeq 2500 platform was used to sequence the sample libraries on 2 × 150 bp chemistry to generate ~ 3Gb of data per sample.

#### Metagenome analysis

Reads obtained from Illumina platform were quality checked using FastQC v0.11.9, and filtered to remove sequencing adapter as well as low-quality bases using Trimmomatic v0.36. Clean reads thus obtained were used for de novo assembly of data. Scaffold generation was done using Metaspades and CLC genomics workbench at default parameters for ABS1 and ABSLW samples. While Metaspades uses de Bruijin graph, CLC genomic workbench uses overlap layout consensus for de novo assembly of reads. The scaffolds thus generated were subject to gene prediction using Prodigal (v2.6.3) followed by diversity analysis using Kaiju [49] which is a very sensitive taxonomy classification tool. It classifies the sequence at protein level using greedy mode. However, functional annotation against COG, KEGG, Pfam, FIG, and GO database was obtained using Cognizer [50] which uses novel-directed search strategy to reduce the computational time.

Ecological distance matrices were calculated and used in this study to find out all the canonical macro-ecological species diversity. To find out the probability that two randomly sampled organisms belong to the same species, Simpson diversity index (D) was calculated [51] according to the following:

1 $$\frac{\sum_i{n}_i\left({n}_i-1\right)}{N\left(N-1\right)}$$

where *n*_*i*_ is the abundance of *i*th species and *N* is the total number of individual present.

Species richness and dominance were calculated in this study via Shannon’s diversity index (*H*) [52] according to the following:

2 $$\sum\nolimits_i\left(\frac{n_i}{N}\ {\log}_2\left(\frac{n_i}{N}\right)\right)$$

where *n*_*i*_ is the abundance of *i*th species and *N* is the total number of individual present.

In an attempt to find out species richness which is independent of sample size, Menhinick index has been calculated [53].

3 $$\frac{S}{\sqrt{\sum_i{n}_i}}$$

Calculation of Buzas and Gibson’s index [54] helped decipher conservation model and observe the trends of changes in an ecosystem.

4 $$\frac{e^{-{\sum}_i\left(\frac{n_i}{N}-\ln \left(\frac{n_i}{N}\right)\right)}}{S}$$

Berger-Parker dominance index [55] is simple mathematical expression relating species richness and evenness.

5 $$\frac{n_{max}}{N}$$

Margalef’s diversity index [56] is a species richness index. Many species richness measures are strongly dependent on sampling effort. The greater the sampling effort, higher the index value. Thus, Margalef’s diversity index considers the number of taxa and total number of individuals.

6 $$\frac{S-1}{\ln N}$$

#### Annotation against CAZy

The family of glycoside hydrolases (GH) is present in the CAZy database (www.cazy.org) which is a database of Carbohydrate Active Enzymes or CAZymes [57]. Henceforth, the CAZy database was used to analyze the GH families present in samples using dbCAN [58].

## Results

### Physicochemical parameters

Soil pH is an indicator of the soil’s acidity or alkalinity. The physicochemical parameters of the soil revealed the pH of the soil to be in the range of 6.25±0.14. Organic carbon is the amount of soil organic matter [59] where the carbon content of the soil was found to be around 1.20 ±0.03; this could also be an essential factor to indicate the abundance of Betaproteobacteria in the soil as elucidated from our study. Nitrogen, i.e., significantly important to plant growth [60] was also checked, and it was found to be around 0.10±0.015. This could facilitate the growth of *Firmicutes *in the soil as they have been reported to play a role in nitrate metabolism [61]. However, the pH of water was found to be neutral around 7.12±0.07, indicating an ideal environment for bacterial growth. Further, conductivity, was found to be around 204.3±0.76 μS cm^-1^, a factor used to gauge the degree of mineralization [62]. Overall, Physicochemical parameters are significant because its disparity can bring about changes in the microbial community [63].

### Taxonomic annotation using standalone Kaiju

**Fig. 1:**
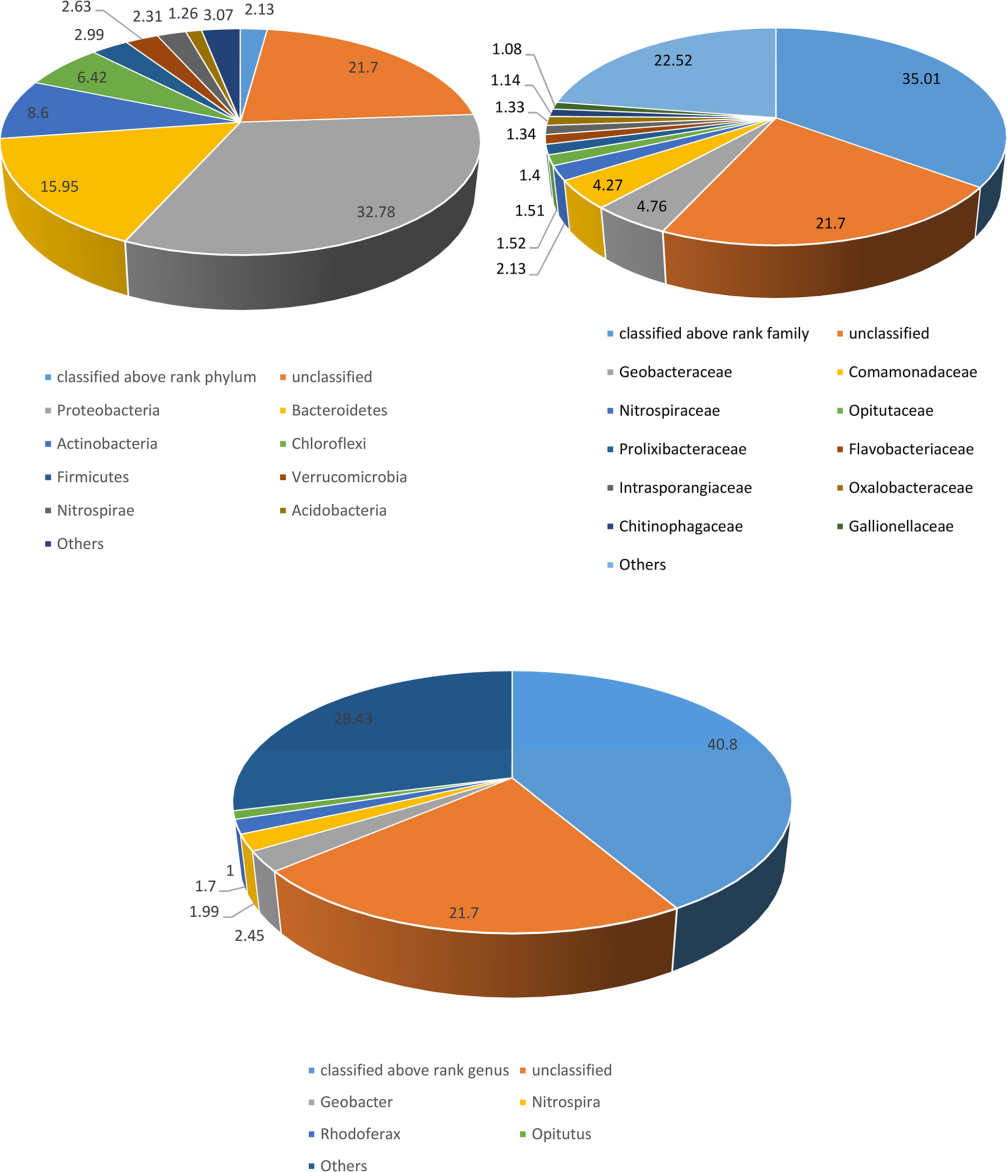
The figure depicts the abundance of bacterial communities of ABS1 sample at different taxonomic units (Phylum Family and Genus). Lesser than 1% of the distribution were together labeled as "Others"

ABS1 taxonomic hits distribution shows Proteobacteria as the most abundant phylum represented by 78,597 genes. Whereas at class level, Betaproteobacteria and Deltaproteobacteria were found to be most abundant represented by 39,405 and 19,928 genes, respectively. At order level, taxonomic hits distribution showed 20,510 genes for Burkholderiales followed by 14,129 for Desulfuromonadales. The taxonomic distribution of genes for the bacterial family revealed 11,413 gene hits on Geobacteraceae followed by 10227 on Commonadaceae. However, at the genus level, there was 5864 gene hits on *Geobacter* followed by 4775 on *Nitrospira*. At the species level the taxonomic hit distribution showed 10,279 gene hits of *Bacteroidetes bacterium GWB2_41_8* followed by 6135 gene hits on *Chloroflexi bacterium* (Fig. 1, Supplementary Figure 1)

**Fig. 2:**
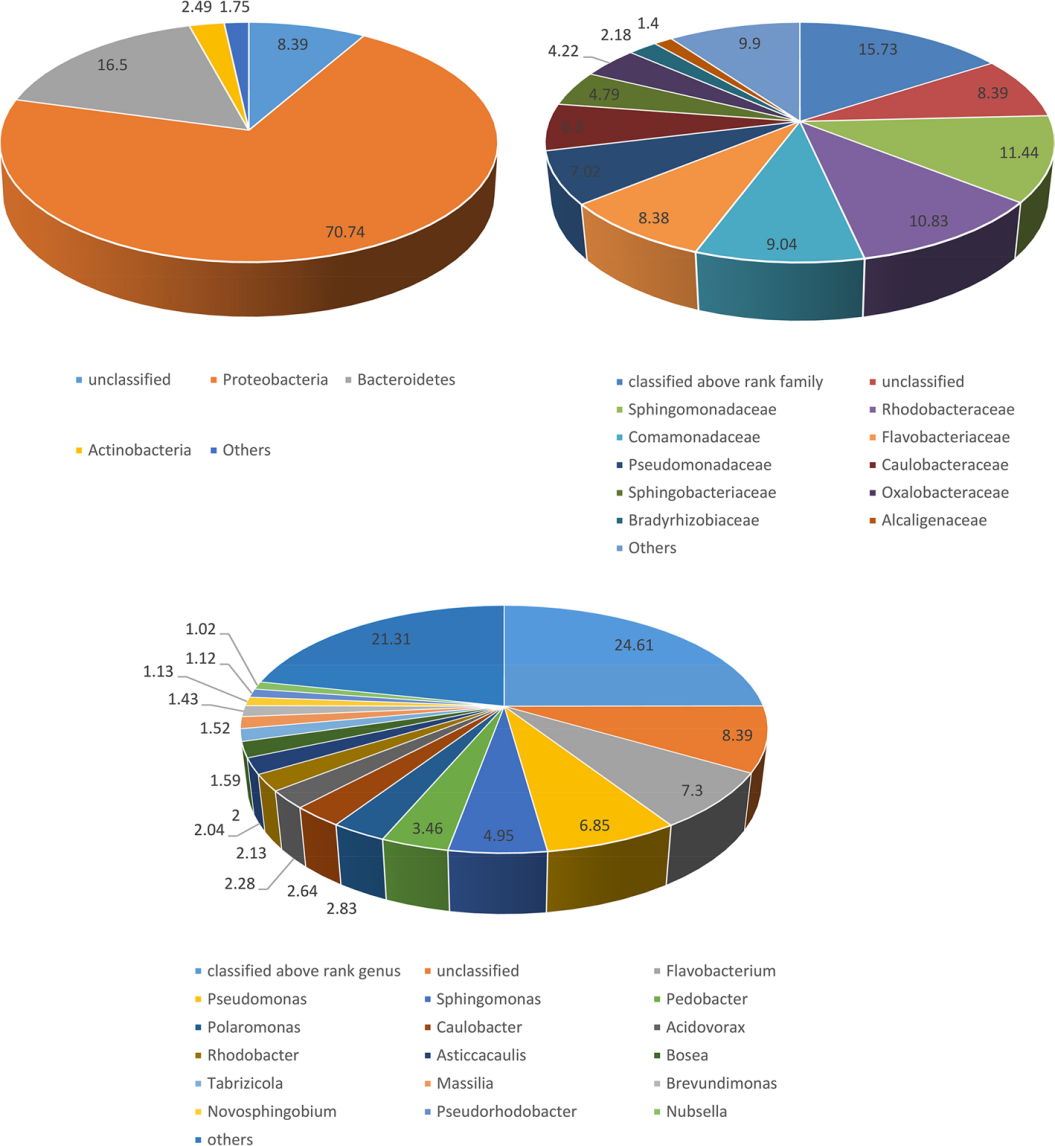
The figure depicts the abundance of bacterial communities of ABSLW sample at different taxonomic units (Phylum Family and Genus). Lesser than 1% of the distribution were together labeled as "Others"

Taxonomic hit distribution of ABSLW shows Proteobacteria as most abundant phylum represented by 104,745 genes, with 62,779 gene hits of Alphaproteobacteria followed by 26,921 hits of Betaproteobacteria at class level. In the bacterial order distribution, taxonomic hits showed 25,406 gene hits of Burkholderailes followed by 22,912 of Sphingomonadales. The taxonomic distribution of gene hits for the bacterial family revealed 16,940 gene hits on Sphingomonadaceae followed by 16,038 Rhodobacteraceae. In the genus level, there was 10,812 gene hits of *Flavobacterium* followed by 10,141 on *Pseudomonas*. At the species level, the taxonomic hit distribution showed 3211 gene hits of *Sphingomonadaceae PASS1* followed by 1905 gene hits of *Oxalobacteraceae* (Fig. 2, Supplementary Figure 2).

### COG functional category hit distribution

The COG database is an endeavor to categorize proteins [64]. The COG functional analysis of ABS1 showed 130,191 genes were assigned to COG where maximum genes, i.e., 14,175 genes, were found to belong to general function prediction only (S1). COG functional analysis of ABSLW shows 104,334 genes assigned to COG, where 10,667 genes were belonging to amino acid transport and metabolism and 11,004 genes falling into the category of general function prediction only (S6). The COG classification also depicted the prevalence of the functions associated with amino acid transport and metabolism, energy production and conversion, general function prediction only, carbohydrate transport and metabolism, lipid transport and metabolism, replication, recombination, and repair, signal transduction mechanisms, translation ribosomal structure and biogenesis, post-translational modification, protein turnover, and chaperones in both the samples.

### KEGG functional category hit distribution

KEGG is a collection of databases that help to predict the different metabolic pathways of a biological system [65]. In ABS1 (S4), functional analysis showed that from a total of 239,754 genes, 160,268 genes were assigned to KEGG classes. Whereas, in the case of ABSLW (S9), KEGG functional analysis showed that from a total of 148,066 genes 122,911 genes were assigned to KEGG classes. In both the cases, the majority of KOs comprised of metabolism category. ABS1 samples revealed a greater involvement of two component systems, cell cycle response regulator, and cell cycle response regulator. However, methyl-accepting chemotaxis protein, ATP-binding cassette, histidine kinase, and RNA polymerase sigma 70 factor were also some of the abundant terms associated with KO of the ABS1 sample. The most abundant KEGG term in ABSLW was found to be iron complex outer membrane receptor protein. Some of the terms associated with the ABSLW sample were also methyl-accepting chemotaxis protein, acyl CoA dehydrogenase, *N*-acetylmuramayl-L-alanine amidase, and ATP-binding cassete. The analysis of the KEGG pathway showed diverse pathways and mechanisms that the microbes thriving in this lake use as a survival tactic to endure the extreme conditions in this region.

### Pfam functional category hit distribution

Pfam is a useful annotation tool for categorizing protein families [66]. Analysis of ABS1 showed that 140,559 genes were assigned with different PFAM domains (S5). Likewise when ABSLW sample was analysed it resulted in assignment of PFAM domains to 111,508 genes, (S10).

### FIGfam functional category hit distribution

Figfams are a set of proteins that share a common function [67]. FIGfams functional analysis of ABS1 (S2) showed that from total of 239,754 genes, 84,544 genes were assigned with different FIG classes. On the other hand 62,074 genes were assigned to different FIG classes for ABSLW (S7).

### Gene Ontology (GO) functional category hit distribution

The Gene Ontology project represents gene product properties [68]. Henceforth, GO functional analysis was carried out for ABS1 and ABSLW samples. It resulted in assignment of 181,375 and 143,201 genes with GO classes, for ABS1 and ABSLW respectively (S3,S8).

### Alpha (α) diversity index

The Simpson index represents the species diversity in a particular habitat. According to our results, the Simpson index was 0.022 (water) and 0.16 (soil). This index represents higher diversity in the habitat. Simpson’s reciprocal index for water and soil samples was 47 and 6.3, respectively. The Shannon index calculated from water and soil samples was 4.2 and 3, respectively, suggesting an even distribution of species. The Menhinick index for both water (.41) and soil (.054) indicates a richness of species. Here, in our results, Buzas and Gibson’s index were found to be.76 and.8, respectively, for water and soil samples, indicating that the species in almost both the samples were evenly distributed. The Berger Parker dominance of the water sample has an index of 0.077, and the soil sample has an index of 0.3, which suggests the samples have higher richness and more abundance. Margalef’s index, in the water sample (7.8) and in the soil sample (.86), signifies abundant diversity (Table 1).

**Table 1:** The table shows the alpha diversity index of water and soil of Samiti lake

Alpha Diversity Index	Equation	Water	Soil
Simpson Index	$\frac{\sum_{\textrm{i}}{\textrm{n}}_{\textrm{i}}\left({\textrm{n}}_{\textrm{i}}-1\right)}{\textrm{N}\left(\textrm{N}-1\right)}$	0.022	0.16
Reciprocal Simpson Index	$\frac{1}{\left(\frac{\sum_{\textrm{i}}{\textrm{n}}_{\textrm{i}}^2}{{\textrm{N}}^2}\right)}$	47	6.3
Shannon Index	$\sum\limits_{\textrm{i}}\left(\frac{{\textrm{n}}_{\textrm{i}}}{\textrm{N}}\ {\log}_2\left(\frac{{\textrm{n}}_{\textrm{i}}}{\textrm{N}}\right)\right)$	4.2	3
Menhinick Index	$\frac{\textrm{S}}{\sqrt{\sum_{\textrm{i}}{\textrm{n}}_{\textrm{i}}}}$	0.41	0.054
Buzas and Gibson's Index	$\frac{{\textrm{e}}^{-{\sum}_{\textrm{i}}\left(\frac{{\textrm{n}}_{\textrm{i}}}{\textrm{N}}-\ln \left(\frac{{\textrm{n}}_{\textrm{i}}}{\textrm{N}}\right)\right)}}{\textrm{S}}$	0.76	0.8
Berger-Parker Dominance Index	$\frac{{\textrm{n}}_{\textrm{max}}}{\textrm{N}}$	0.077	0.3
Margalef Richness Index	$\frac{\textrm{S}-1}{\ln \textrm{N}}$	7.8	0.86

### Glycoside hydrolase

**Fig. 3:**
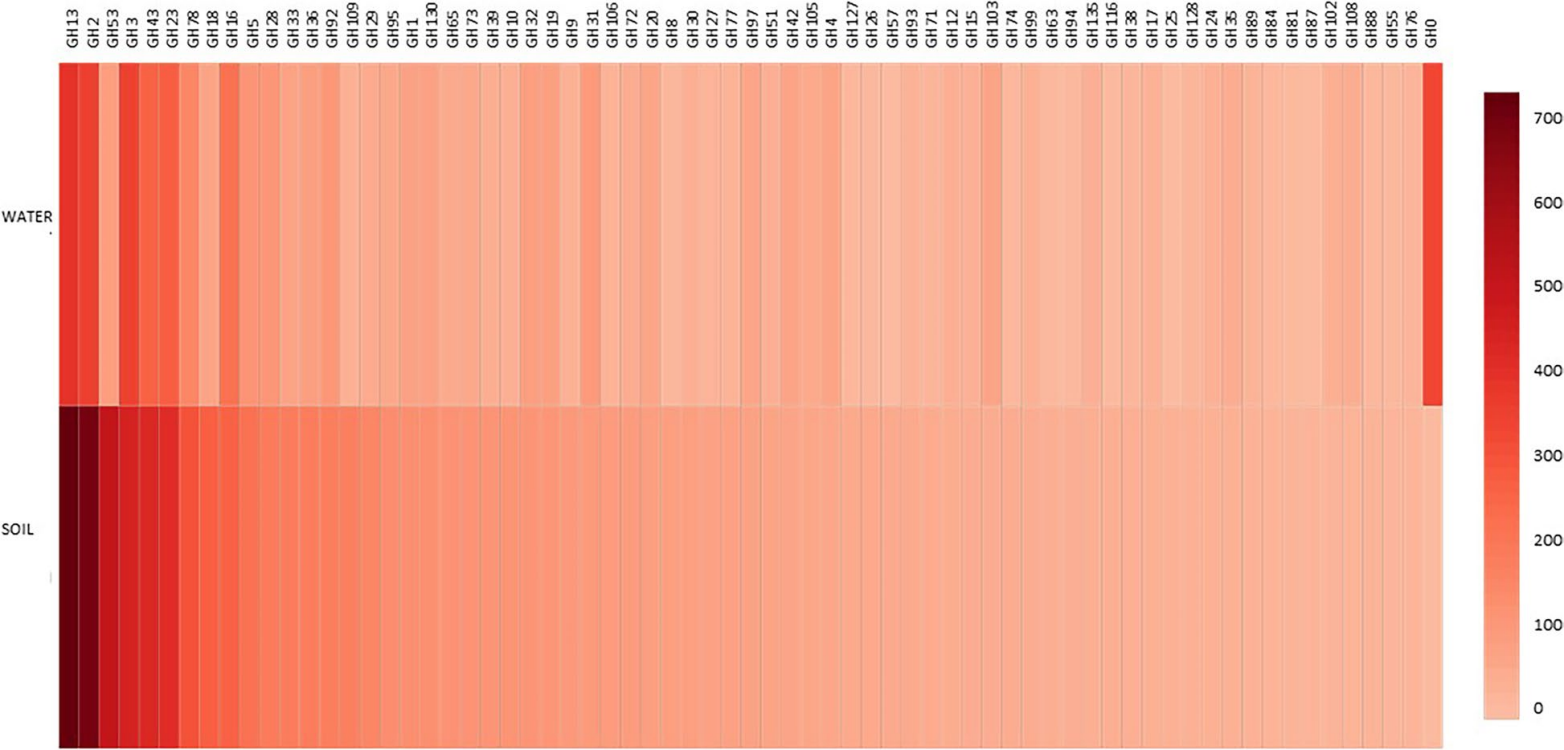
The heat map depicts the presence of glycoside hydrolases in ABS1 and ABSLW samples

Our study shows an abundance of GH13, GH2, GH3, GH43, and GH23 in both the soil and water samples in high numbers as is evident from the (Fig. 3); however, the presence of GH53 was abundant in ABS1 compared to ABSLW.

## Discussion

Our study has indicated an abundance of Proteobacteria in this region, which is very similar to the reports by Gangwar et al. [21] who revealed a predominance of Proteobacteria, in Western Himalayas. Reports from Pangong lake, Tsomgo Lake [9, 69], some of the high-altitude Himalayan lakes, Alpine lakes of Hengduan Mountain [70], and Sayram lake [71] have also reported the abundance of Proteobacteria. Many members of *Proteobacteria* are involved in the metabolism of sulphate and nitrate [61]. Proteobacteria have been reported to play significant roles in carbon sequestration and nitrogen cycling [72]. The abundance of AlphaProteobacteria in water sample is well justified as it requires minimal amount of nutrients [73]. However, the presence of Betaproteobacteria in the soil sample elucidates the fact that it extensively utilizes the nutrient of the soil. The abundance of Firmicutes also sheds light on its role in nitrate, methane, and sulphate metabolism as has been reported by Haldar et al. [61].

Studies conducted widely over polar and non-polar glaciers have also reported the presence of genera like *Proteobacteria*, *Actinobacteria*, and *Flavobacteria* [74]. At the genus level *Flavobacterium* was the most abundant taxon in water. *Flavobacteria* have been reported to play an imperative role mineralizing poorly degradable macronutrients and providing their surrounding environments with carbon flux regulators [75]. *Genus Flavobacterium* are considered as chief mineralizers of organic matter, numerous reports of their isolation from soil [76], water [77], Antarctic regions [78], and glacier samples [79] have been described. Further, it has been reported that many members of *Geobacter* play a role in carbon and metals cycling in aquatic sediments and also help in the bioremediation of metal-contaminated groundwater and harvesting of electricity [80, 81]. They have been found to play significant roles in both pristine and contaminated subsurface environments [82, 83], also helping in bioremediation [84]. Moreover, some *Geobacter* species have been reported to help in sulfur reduction, [85] where most of the mechanisms of sulfur reduction relates to elemental sulfur being converted to H_2_S [86], ultimately, sulfide the ultimate byproduct serves as an electron donor for a wide range of microbial metabolisms [87].

Glycoside hydrolases facilitate carbohydrate degradation and are thus sought after enzyme in different industries [88]. The abundance of GH13 in this lake indicates the predominance of α-amylase (very significant in the hydrolysis of starch and related poly- and oligosaccharides) [89] as its been reported as an essential representative of the GH13 family [89]).  Numerous reports of microbial α-amylases in bioremediation and biorefinery [90, 91] also highlights its importance. The prominence of GH43 also indicates the presence of many enzymes under GH43 involved in the breakdown of pectin and hemicellulose polymers [92]. Another major industrially important enzyme β-galactosidases are glycoside hydrolases (GH) that give galactose molecules by hydrolyzing glycosidic bonds [42]. Galactosidases hold great potential in industrial and biotechnological applications [42]. These enzymes are ubiquitous and have been isolated from diverse and extreme environments [42, 93]. The lake also has the presence of high quantity of GH3, and since this lake is located at a very high altitude, it could also possess psychrophilic and psychrotrophic β-glucosidase. Recent, reports on psychrophilic *Paenibacillus* sp. for production of cold-active β-glucosidase belonging to GH3 family have been elucidated [41]. The abundance of GH2 in this lake predicts that this lake could harbor cold active GH2s, as this lake is located at a very high altitude. The application of numerous cold-active GH2s having potential in alkyl galactopyranosides synthesis and lactose hydrolysis has been reported from *Pseudoalteromonas* sp. 22b and *Pseudoalteromonas haloplanktis* [94, 95]. The presence of GH23, which is very prevalent in cell wall degradation [88] has also been reported in this lake. Further, the abundance of GH53 in the soil sample could facilitate the growth of probiotic strains in this lake as GH53 has been reported to break down Galactooligosaccharides [96]. The unknown richness of this lake may also hold a wealth of biotechnological uses, as earlier studies of the Himalayan range have described [97, 98].

## Conclusion

The presence of varied microbial diversity in this lake predicts a thriving ecosystem, housing different species living in harmony, and building a repository of varied metabolic functions to keep the lake thriving and alive. It also reports the functional characteristics of the microbial diversity, and the different metabolic approaches employed by these microorganisms for their survival in these extreme conditions. This includes different genes involved in defense mechanisms, signal transduction mechanisms, transcription, co enzyme transport, and metabolism that they use to survive at this high-altitude and extreme changing environmental conditions. The abundance of glycoside hydrolases highlights the fact that this lake could be a repository of numerous psychrophilic and psychrotrophic glycoside hydrolases, thus paving the way towards a novel discovery that could be beneficial in bio refinery and bioremediation sectors. However, further studies based on the intricacies of carbohydrate deconstruction, and the mechanism of the microbial community involved in the breakdown of the polymer could provide a breakthrough in CAZyme research. The diverse metabolic pathways depicted through this study gives us an insight into the larger scope of exploration of microbes with bioprospective potential. However, the recent anthropogenic activity has definitely remained a threat to the prevailing ecosystem, and the effect of climate change in recent times has also threatened the microbial diversity of such ecological hotspots. Moreover, the discovery of novel microbes with psychrophilic properties that are involved in xenobiotic degradation and catabolism of recalcitrant chemicals could also prove to be a promising feat for designing bioremediation approaches if required.

## Supplementary Information

**Additional file 1: S1.** The table shows functional annotation against COG database for ABS1 sample from Samiti Lake. **S2.** The table shows functional annotation against FIG database for ABS1 sample from Samiti Lake. **S3.** The table shows functional annotation against GO database for ABS1 sample from Samiti Lake. **S4.** The table shows functional annotation against KEGG database for ABS1 sample from Samiti Lake. **S5.** The table shows functional annotation against PFAM database for ABS1 sample from Samiti Lake. **S6.** The table shows functional annotation against COG database for ABSLW sample from Samiti Lake. **S7.** The table shows functional annotation against FIG database for ABSLW sample from Samiti Lake. **S8.** The table shows functional annotation against GO database for ABSLW sample from Samiti Lake. **S9.** The table shows functional annotation against KEGG database for ABSLW sample from Samiti Lake. **S10.** The table shows functional annotation against PFAM database for ABSLW sample from Samiti Lake

**Additional file 2: Figure S1.** The Krona graph shows the phylum Proteobacteria to be highest in ABS1 sample. **Figure S2.** The Krona graph shows the phylum Proteobacteria to be highest in ABSLW sample.

## Data Availability

The datasets generated during and analyzed during the current study are available in NCBI with accession numbers SAMN13671136 and SAMN13671135.

## Abbreviations

GH: Glycoside hydrolases
COG: Cluster of Orthologous Groups of Proteins
KEGG: Kyoto Encyclopedia of Genes and Genomes
Pfam: Protein families
GO: Gene Ontology
