# Supplementary material for: A shotgun approach to explore the bacterial diversity and a brief insight into the glycoside hydrolases of Samiti lake located in the Eastern Himalayas
Source: J Genet Eng Biotechnol. 2022 Dec 5;20:162. doi: 10.1186/s43141-022-00444-y (PMC9723087; doi:10.1186/s43141-022-00444-y)
Supplement: Supplementary file 2 — Additional file 2: Figure S1. The Krona graph shows the phylum Proteobacteria to be highest in ABS1 sample. Figure S2. The Krona graph shows the phylum Proteobacteria to be highest in ABSLW sample. [file 43141_2022_444_MOESM2_ESM.docx]

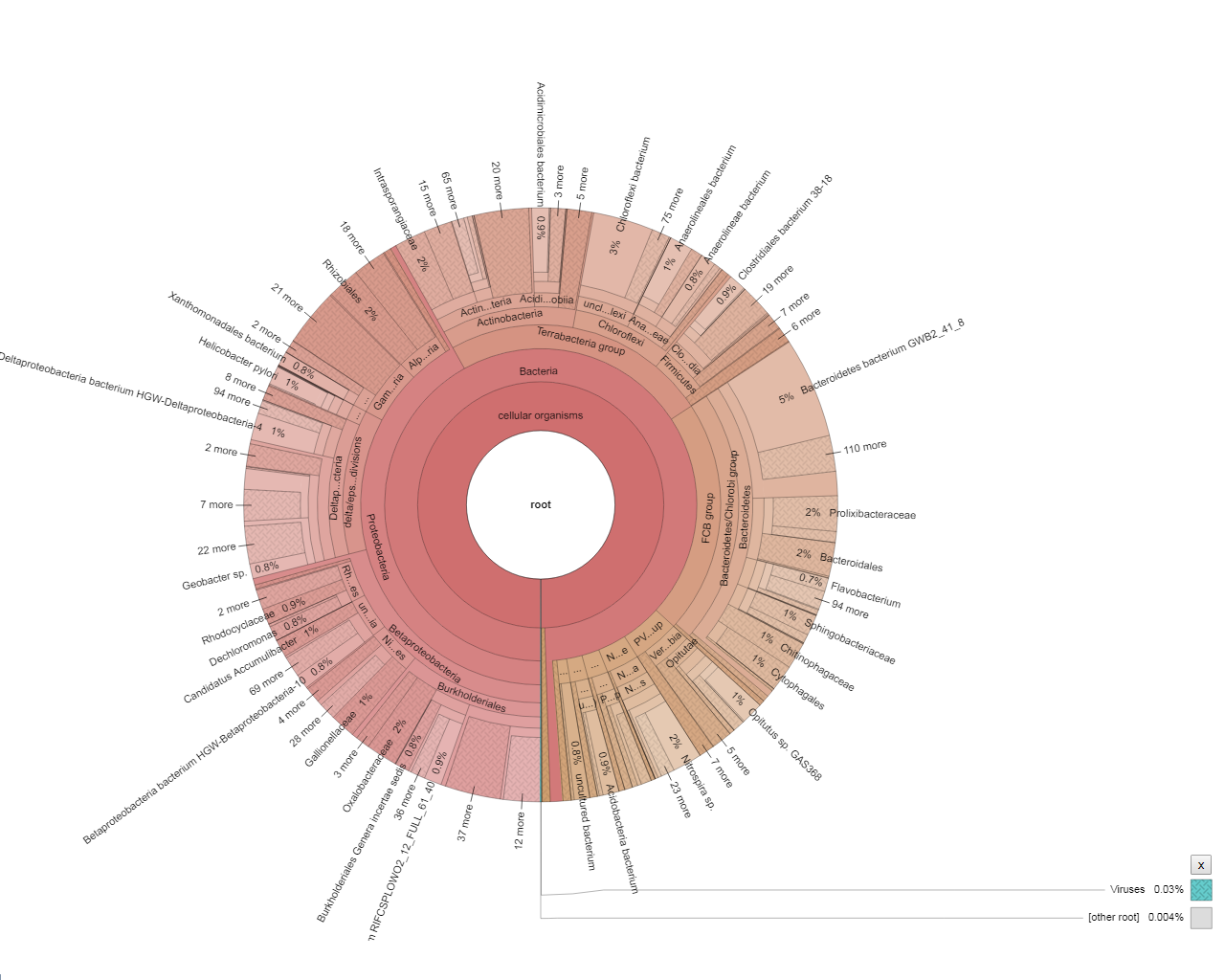


Supplementary Figure 1 The Krona graph shows the phylum Proteobacteria to be highest in ABS1 sample


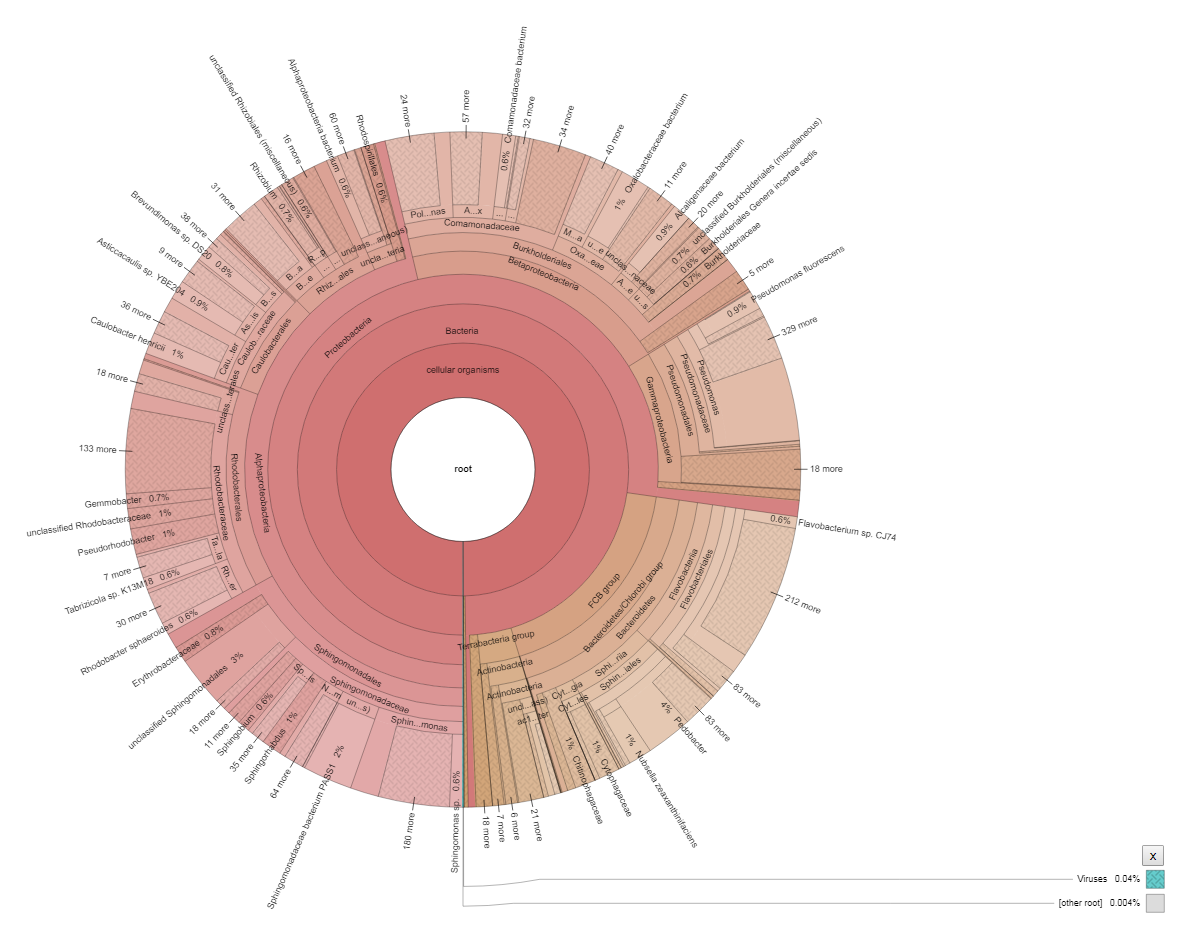


Supplementary Figure 2 The Krona graph shows the phylum Proteobacteria to be highest in ABSLW sample.
